# 2314. Association Between Behavior and Risk of COVID-19 in a Cohort of Healthcare Personnel

**DOI:** 10.1093/ofid/ofad500.1936

**Published:** 2023-11-27

**Authors:** Holly Shoemaker, Haojia Li, Yue Zhang, Jeanmarie Mayer, Michael Rubin, Morgan M Millar, Per H Gesteland, Andrew T Pavia, Lindsay T Keegan, Jordan Braunfeld, Kristina Stratford, Matthew H Samore

**Affiliations:** University of Utah, Salt Lake City, Utah; University of Utah, Salt Lake City, Utah; University of Utah, Salt Lake City, Utah; University of Utah, Salt Lake City, Utah; University of Utah, Salt Lake City, Utah; University of Utah, Salt Lake City, Utah; University of Utah School of Medicine, Salt Lake City, Utah; University of Utah, Salt Lake City, Utah; University of Utah, Salt Lake City, Utah; University of Utah, Salt Lake City, Utah; University of Utah, Salt Lake City, Utah; University of Utah, Salt Lake City, Utah

## Abstract

**Background:**

Social behaviors are associated with COVID-19 infection. Most studies assessing exposure risk from daily activities were performed in the general population. Less is known about the impact of social behavior on the risk of COVID-19 among healthcare personnel (HCP), who have a higher risk of exposure at work and more awareness of prevention measures than the general population.

**Methods:**

We conducted a prospective cohort study, with monthly surveys from Dec 2021 to June 2022 of HCP at an academic healthcare system. Each survey asked HCP if they had been tested for SARS-CoV-2, their results, and if they had engaged in any of 9 common social activities outside of work (see figure). A composite social activities exposure measure was defined as the count of “Yes” to the social activities at the previous survey, with a possible range from 0-9. Similarity of current overall social behavior compared to pre-pandemic was also reported on a scale of 0-10, with 10 reflecting the same level as pre-pandemic. We employed mixed effect logistic regression, weighted for dropout using covariate balancing propensity scores, to estimate the association between previously reported social behavior and activities and testing positive for SARS-CoV-2 after adjusting for covariates (see table).

**Results:**

Recruitment emails were sent to 10,321 HCP, with 1,802 (17.5%) consenting to participate and 1,302 (72.3%) completing 2 or more surveys. 981 HCP reported being tested for SARS-CoV-2 with at least one positive test reported by 277 HCP (28.2%). Engaging in overall social behavior more similar to pre-pandemic levels was found to be statistically significantly associated with an increased likelihood of testing positive for SARS-CoV-2 (OR 1.32, 95% CI 1.11-1.56). However, while there was a trend to testing positive with an increased number of social activities reported at the previous survey, it was not significant (OR 1.06, 95% CI 0.95-1.18).

Temporal Trends of Social Activities in Healthcare Workers

**Figure:**
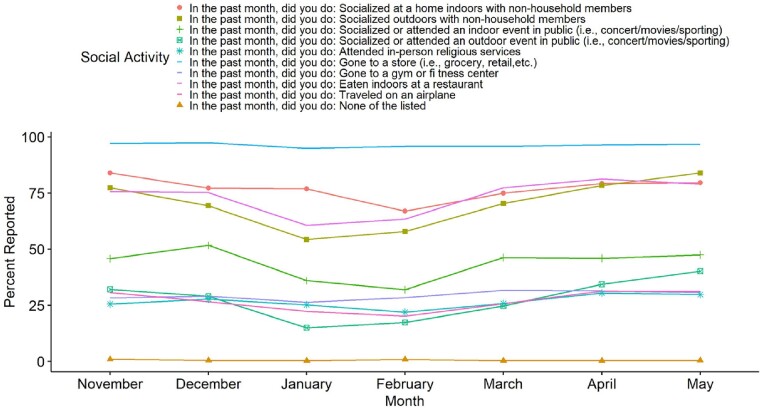

Reported social activities of healthcare workers at an academic healthcare system from late November 2021 to May 2022.

Association Between Social Activity Measures and Positive COVID-19 Test

**Figure:**
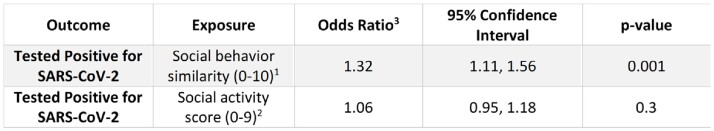

1. Similarity of social behavior at the previous survey with pre-pandemic behavior reported on a scale of 0-10, with 10 reflecting the same level of activity as pre-pandemic. 2. Count of “Yes” to the social activities at the previous survey, with a possible range from 0-9. 3. Covariates adjusted: calendar month, age, gender, clinic role, working location, general health, comorbidities, household status, illnesses reported at the previous survey, time since last vaccine, and time since the previous survey.

**Conclusion:**

This study, which included the Omicron surge, saw HCP participation in a range of social activities. Reporting levels of social activity similar to pre-pandemic levels was associated with an increased risk of subsequent COVID-19. This feedback is important for HCP consideration of exposures outside of work.

**Disclosures:**

**Jeanmarie Mayer, MD**, GlaxoSmithKline: Stocks/Bonds **Andrew T. Pavia, MD**, GlaxoSmith Kline: Advisor/Consultant|Sanofi: Advisor/Consultant

